# Evolution of replication origins in vertebrate genomes: rapid turnover despite selective constraints

**DOI:** 10.1093/nar/gkz182

**Published:** 2019-03-27

**Authors:** Florian Massip, Marc Laurent, Caroline Brossas, José Miguel Fernández-Justel, María Gómez, Marie-Noelle Prioleau, Laurent Duret, Franck Picard

**Affiliations:** 1 Université de Lyon, Université Lyon 1, CNRS, Laboratoire de Biométrie et Biologie Evolutive UMR 5558, Villleurbanne, France; 2 Berlin Institute for Medical Systems Biology, Max Delbrueck Center for Molecular Medicine, Berlin, Germany; 3 Institut Jacques Monod, CNRS UMR7592, Université Paris Diderot, Equipe Labellisée Association pour la Recherche sur le Cancer, Paris, France; 4 Centro de Biología Molecular Severo Ochoa CBMSO (CSIC/UAM). Nicolás Cabrera 1, 28049 Madrid, Spain

## Abstract

The replication program of vertebrate genomes is driven by the chromosomal distribution and timing of activation of tens of thousands of replication origins. Genome-wide studies have shown the association of origins with promoters and CpG islands, and their enrichment in G-quadruplex motifs (G4). However, the genetic determinants driving their activity remain poorly understood. To gain insight on the constraints operating on origins, we conducted the first evolutionary comparison of origins across vertebrates. We generated a genome-wide map of chicken origins (the first of a bird genome), and performed a comparison with human and mouse maps. The analysis of intra-species polymorphism revealed a strong depletion of genetic diversity at the core of replication initiation loci. This depletion is not linked to the presence of G4 motifs, promoters or CpG islands. In contrast, we show that origins experienced a rapid turnover during vertebrate evolution, since pairwise comparisons of origin maps revealed that <24% of them are conserved among vertebrates. This study unravels the existence of a novel determinant of origins, the precise functional role of which remains to be determined. Despite the importance of replication initiation for the fitness of organisms, the distribution of origins along vertebrate chromosomes is highly flexible.

During each cell cycle, the genome must be accurately replicated to ensure the faithful transmission of the genetic material to daughter cells. In vertebrates, as in many other eukaryotes, this fundamental process is initiated at specific sites, called replication origins (1). Several studies in humans and mice have shown that their genomes consist of large domains (from 200 kb to 2 Mb) that are replicated at different time points of S-phase (2–4). This spatio-temporal program of genome replication is driven by the chromosomal distribution of replication origins and by their timing of activation. Thanks to the development of new techniques, based on the purification of short nascent DNA sequences (SNS), it has been possible to obtain genome-wide maps of replication origins at the kb resolution in human (5–7), mouse (8,9), Drosophila (10) nematode (11,12) and the human parasite *Leishmania major* (13). In humans and mice, 65 000–250 000 origins have been identified (6–9), which reflects the number of origins that are active in a population of cells. However, autoradiographic evaluation of the distance between two active origins (14), has led to the estimate that, at most, 30 000 origins are actually activated in each cell cycle in mammals. Furthermore, the comparison of five different human cell types has uncovered substantial plasticity in the spatial program of replication : ∼10–15% of replication origins are cell-type-specific, whereas ∼35–60% are constitutively active (6,7).

Despite these important advances in the characterization of replication origins, the genetic determinants of their activity are still poorly understood. In both human and mice, replication origins are strongly enriched in transcription start sites (TSSs) and CpG islands (CGIs) (5,7,8,15). They are also associated with specific epigenetic marks (7,16), depending on the progression of the cell cycle. Mammalian replication origins are enriched in G-rich motifs that are capable of forming DNA secondary structures called G-quadruplexes (G4) (6,7,17,18) and there is experimental evidence that G4 elements can directly contribute to the function of origins (19). However, the presence of a G4 motif is not sufficient for origin activity (19), and 50% of these motifs are found outside of known origins (18), implying that other genetic elements must contribute to the origin activity.

To gain insight into the selective constraints that operate at the location of replication origins and the genetic elements that determine their activity, we decided to investigate the evolution of replication origins in vertebrates. For this purpose, we mapped origins in the chicken genome. We chose chicken because the evolutionary distance between mammals and birds is such that only functionally constrained elements are conserved between their genomes (20). Here, we report a comparative analysis of replication origin landscapes across mammals and birds, and an investigation of selective constraints that act on their sequences, based both on an inter-species comparison and on an analysis of intra-species genetic polymorphism.

The polymorphism analysis demonstrates that the locus of maximal SNS enrichment within replication origins contains functionally important sequence elements that are clearly subject to purifying selection and distinct from G4 motifs. However, inter-species comparisons reveals a poor conservation of these elements on a larger evolutionary scale and shows that the location of replication origins evolves rapidly. Furthermore, the chromosomal distribution of origins in mice is very different from that observed in human and chicken. These observations indicate that despite the importance of replication initiation activity for the fitness of organisms, the distribution of replication origins along chromosomes is highly flexible.

## MATERIALS AND METHODS

### SNS data generation

Short nascent strands were purified as previously described (21). We pooled fractions 15–20 containing single-stranded DNA molecules with a size between 1.5 and 2.5 kb in size. We used 500 U of a custom-made λ-exonuclease (ThermoFisher Scientific, 50 U ml^−1^, ref: EN056B1C002) for each preparation. For the genome-wide mapping of origins, six SNS preparations were obtained independently from 10^8^ cells each and then pooled. SNS were double-stranded by random priming with the Klenow exo-polymerase (#EP0421, Thermo Fisher Scientific) and random primers (#48190011, ThermoFisher Scientific). Adjacent strands were then ligated with Taq DNA Ligase (M0208L, Biolabs). RNA primers were hydrolysed for 30 min at 37°C with 2.5 M NaOH. The reaction was then neutralized with 2.5 M acetic acid, and SNS were purified by phenol chloroform extraction and recovered after ethanol precipitation and resuspension in TE buffer. SNS molecules were quantified with the QBit dsDNA HS assay kit (Q32854, Thermo Fisher Scientific) and 225 ng of double-stranded material was used for fragmentation using a Covaris apparatus with a 50-μl microTUBE-50 AFA Fiber screw-cap tune (PN 520166, Covaris) and a M220 Holder XTU (PN 500488, Covaris). We used a sonication program with 75 W peak incident power, 10% duty factor and 200 cycles per burst running at 4°C for 195 s to obtain molecules with a size of approximately 180 bp. The library was constructed with the NEBNext©Ultra™ II DNA Library Prep Kit for Illumina© (NEB #E7645S) following the manufacturer’s instructions with some minor modifications. For the adaptor ligation, undiluted adaptor and no size selection were used. The library amplification was performed using NEBNext ©Multiplex Oligos for Illumina© (NEB #E7335S) with NEBNext index 4 primer (#E7314A) and the NEBNext Universal PCR Primer (#E6861A) with three PCR cycles. Library purification was performed with the SPRIselect Reagent kit (Beckman coulter #B23317), and the final elution step was reduced to 20 μl of 0.1× TE. The mean size of the library molecules determined on an Agilent Bioanalyser High Sensitivity DNA chip (5067–4626, Agilent technologies) was 550 bp. Sequencing was performed on a NextSeq 500 Illumina sequencer with a High Output 150 cycles flow cell (paired-end reads of 75 bp) according to standard procedures. A total of 24 614 M clusters were generated and provided 49 228 M of reads. All Illumina sequencing runs were performed at the GENOM’IC facility of the Cochin Institute.

### SNS data meta-analysis

For human and mouse data, we reanalyzed SNS replication data generated in previous studies. Human H9, IMR90, HeLa and iPS data were from (6) and K562 data from (7). The analysis of mouse data was performed on data from (8) and (9). For the (8) dataset, we kept only two of the three replicates of the experiment due to the apparent low specificity of data collected for the third replicate (as confirmed by personal communication with the authors).

### Origin detection

We reanalysed all SNS data using the same bioinformatics procedure. Reads were mapped using Bowtie2 (22) on hg19, mm10 and gal5 genomes. We excluded reads with quality scores lower than 40, as well as those mapped to non-mappable regions of each genome. To detect origins, we used scanning windows to detect significant reads enrichment along chromosomes (7), with a controlled multiple error rate of 1%. This method was previously validated by a detailed comparison with other molecular approaches. Indeed, two-thirds of the origins detected with our method were confirmed by bubble-trapping (7), and more recently, 90% were confirmed by MCM7 chip-seq peaks in an independent study (23) on HeLa cells. Also, initiation-site sequencing (ini-seq) has been shown to have the highest and most specific concordance with sites identified by our method (24). To increase the resolution of our detections we used smaller windows (500 bp instead of 2 kb (7)), as we found that this window size better reflects the outcome of the SNS experiment. To study origins at the base pair scale, we defined ‘SNS peaks’ as the position inside each origin for which the read accumulation profile was maximal. We computed this maximum on a smoothed read accumulation profile using a Gaussian kernel transformation with a bandwidth of $\sqrt{500}$ bp.

### Databases

CGI annotations were downloaded from the UCSC database (http://genome.ucsc.edu/index.html, (25)) and TSSs from the Eukaryotic Promoter Database (EPD: http://epd.vital-it.ch/) (26) for human and mouse, and from the Ensembl website (http://www.ensembl.org/index.html) (27) for chicken.

### Detection of G-quadruplex motifs

G-quadruplex motif positions were detected on both strands based on the simple motif ${{\texttt G}}_3 {\texttt {N}}_{1-7} {\texttt {G}}_3 {\texttt {N}}_{1-7} {\texttt {G}}_3 {\texttt {N}}_{1-7} {\texttt {G}}_3$, using a dedicated python script (https://github.com/dariober/bioinformatics-cafe/tree/master/fastaRegexFinder).

### Resampling procedure

To correct for the higher sequencing depth of the chicken SNS experiment, we pooled together all human H9 replicates on the one hand, and all mouse mESC replicates, on the other hand, to generate one large human and one large mouse sample. These two datasets showed a similar sequencing depth of 28 reads/kb in human and 24 reads/kb in mouse. We randomly sampled ∼20% of the chicken SNS data to generate three independent datasets in the chicken genome with a sequencing depth of 28 reads/kb, comparable to the datasets in human and mouse. To limit the inter-cell line variability, we focused on the human H9 cell line, because it is the closest cell type to mESCs in mouse for which SNS data are available.

### Cell type specificity

To control our analysis of origin cell type specificity (Figure 6), we first pooled and subsampled reads of different replicates of each cell type experiment to generate five datasets (one for each cell line) with en equal sequencing depth (32 million mapped reads) and detected origins in these datasets. The sequencing depth of these datasets was much lower than the one previously described such that in the new H9 set, we retrieved 80% of the H9 origins set only.

### Polymorphism analysis

We collected human polymorphism data from the 1000 Genomes project (phase3): 3.6 million indels and 84 million SNPs, polarized using great ape genomes as outgroups (28). The base-specific SNP density shown in Figure 4 and S4 is defined as the number of observed *x* → *y* variants divided by the number of *x* bases, where *x* and *y* correspond respectively to the ancestral and derived alleles (${\texttt {A}}$, ${\texttt {C}}$, ${\texttt {G}}$ or ${\texttt {T}}$). Human SNPs were categorized according to the frequency of the derived allele (DAF) among the 2500 individuals sequenced by the 1000 Genomes project: rare variants: DAF <1%; common variants: DAF >10%. Mouse and chicken polymorphism datasets (17 and 20 millions SNPs, from dbSNP v142 and v147 respectively (29)) were downloaded directly from the UCSC genome browser (25). dbSNP entries originate from several studies, with relatively small sample sizes (<20 individuals). It was therefore not possible to distinguish rare variants from common variants in these two species.

### Clustering genomic segments based on kmer composition

We sampled random genomic segments of 40 bp in each genome. The number of random segments was the same as the number of origins in each species. Then, the 5-mer frequencies were computed for all random segments. To reduce dimensionality before clustering, we performed dimension reduction based on NMF (non-negative matrix factorization), a modified version of PCA adapted to count data (30). Then, we used *k*-means to cluster random segments into homogeneous compositional groups using NMF principal components. The numbers of axes and clusters were chosen by rule of thumb. This automatic rule selected six axes. We then detected 6 clusters in the random segments for each species (Supplementary Table S3), which we referred to as background clusters because they represent the background compositional heterogeneities that characterize each genome. For each species, we also computed the 5-mer frequencies in replication origins, which were assigned to background clusters by using the maximum a posteriori rule based on their *k*-mer composition (Supplementary Table S3).

### Randomization procedures

For each species, we generated 10 sets of random genomic positions in mappable regions of the genomes. On each chromosome *i, N*_*i*_ random loci were generated from a uniform distribution where *N*_*i*_ is the number of origins on this chromosome. Each such locus was extended to a segment whose length was uniformly drawn in the size distribution of origins of the same chromosome.

### Activity conservation of TSS

To compare TSS conservation to origin conservation, we first extended TSSs on their 5′ side to generate a set of regions with the same size distribution as origins. We then proceeded to a similar conservation analysis on these sets as done for the origins. Random TSSs sets were generated according to the randomization procedure detailed above.

### Homologous SNS read accumulation profiles

To generate homologous SNS read accumulation profiles (Figure 7), we identified the homologs of the SNS peaks in origins overlapping a CGS for all pairwise comparisons. We then computed the number of SNS reads in a window of 3 kb centred on the SNS peak and of its counterpart in the sister genome.

## RESULTS

### Large-scale variations in replication initiation landscapes

To study the evolution of replication initiation landscapes, we generated the first map of replication origins in a bird genome, by sequencing short nascent strands (SNS) in the chicken DT40 cell line (derived from a bursal lymphoma, see Materials and Methods). For a comparison with mammalian origins, we re-analysed all previously published human and mouse SNS datasets (6–9) using the same detection pipeline, with a resolution of 500 bp (see Methods). This method (7) detects genomic segments on which the accumulation of reads is significantly higher than the background, which depends on the global read depth of the experiment (Supplementary Table S1).

In the following comparisons, we were particularly careful to differences in sequencing depth between experiments, since any detection method based on reads enrichment is more sensitive when the global coverage is high (Supplementary Figure S1). Thus, we sub-sampled SNS reads so that all three species reached the same sequencing depth (see Methods section). In human and mouse, SNS datasets are available for different cell lines (human: IMR90, H9, K562, HeLa and iPS; mouse: mESC10, MEFs). To limit differences due to tissue-specific origin activity, evolutionary comparisons are based on origins detected in H9 (human) and mESC10 (mouse) cell lines, both of which correspond to embryonic stem cells.

As expected given the differences in genome sizes (1.2 Gb in chicken versus ∼3 Gb in mammals), we identified fewer origins in chicken (33 500) than in human (155 395) or mouse (205 881) (Supplementary Table S2). However, variations in the number of origins are not entirely explained by genomes size since we observe a 2.5-fold variation in origin density, from 34 origins per Mb in chicken to 84 origins per Mb in mouse (55 origins/Mb in human). Mouse origins are on average shorter (690 bp), than human (791 bp) or chicken origins (897 bp), but the short size of mouse origins might be due to the selection of shorter nascent strand in mouse experiments (0.5–2 kb) compared to chicken (1.5–2.5 kb) and human (1–2 kb) experiments. Overall, replication origins cover 3% (chicken), 5.5% (human) and 4.3% (mouse) of the genome.

**Figure 1.**
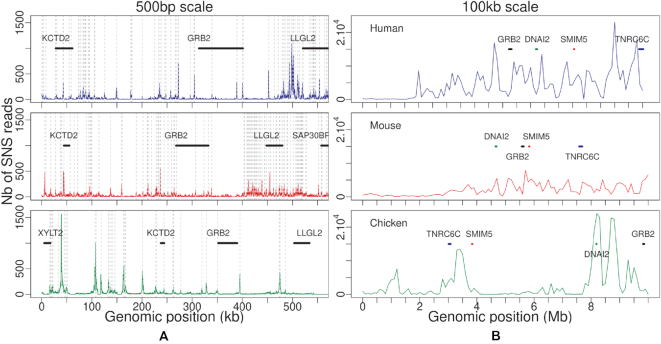
Profile of replication initiation activity along a representative homologous locus in human (top), mouse (middle) and chicken (bottom) genomes. (**A**) Number of SNS reads per 500 bp window in human chr17:73 000 600–73 550 600, mouse chr11:115 376 000–115 926 000, and chicken chr18:10 486 119–11 036 119 regions. (**B**) Number of SNS reads in 100 kb windows in a 10 Mb region centred on the region presented in (A). Vertical grey dotted lines represent detected origins, and several homologous genes are annotated with thick lines.

**Figure 2.**
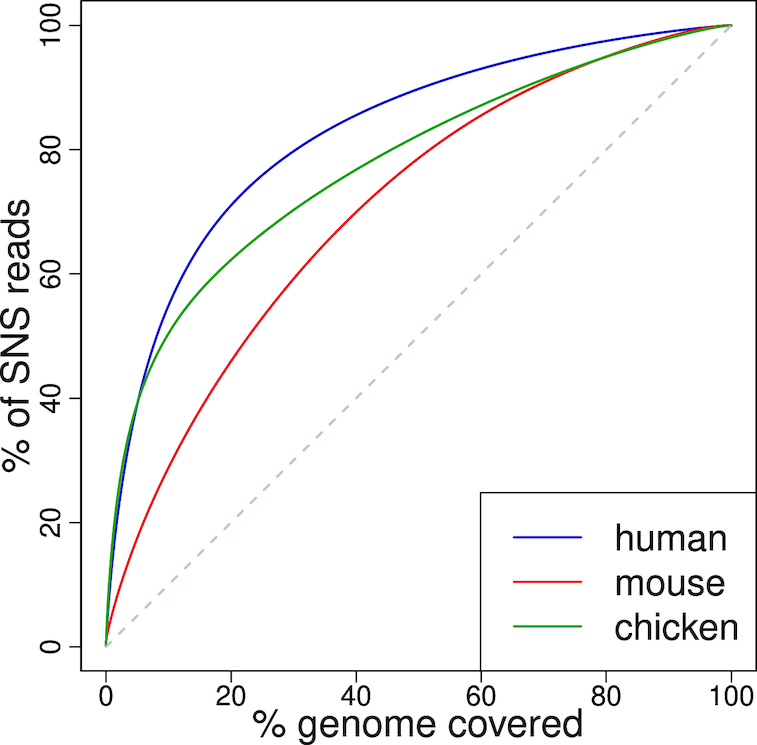
Replication initiation landscapes in vertebrates: Cumulative proportion of SNS reads mapped to the *x* richest 100 kb regions of the genomes. The black broken line corresponds to the expectation under a uniform distribution of reads.

Visual comparison of several homologous regions in the three species revealed that replication initiation landscapes are quite different in mouse compared to human and chicken: we observe many more peaks in mouse, but these peaks are less intense (Figure 1). To extend this comparison over the whole genome, we analysed the distribution of replication initiation activity in 100 kb windows (Figure 2). In mouse, 5% of the genome concentrates 17.5% of the replication initiation activity, compared to 39.1% in human and 39.2% in chicken. Thus, origin activity is more uniformly distributed in mouse than in human and chicken (Figure 2). It should be noted that for both human and mouse, we obtain consistent results with SNS datasets corresponding to distinct cell lines generated by different laboratories (Supplementary Figure S2). Thus, the differences in replication initiation landscapes observed between human and mouse are reproducible, and are not explained by the cell type variability. Interestingly, the more uniform replication initiation landscape that we observe in mouse compared to human coincides with a more uniform GC content distribution (31). In agreement with previous observations in human (5,6), we observe strong positive correlations between the origin density and regional genomic GC content in all three species (Supplementary Figure S3A–C). Indeed, correcting for GC content differences among species strongly reduced the differences between replication landscapes in the three species (Supplementary Figure S3D and E). All these observations indicate that the number of replication origins is not simply dictated by the evolution of genome size and that even within mammals, there are substantial differences in the distribution of replication initiation activity along chromosomes.

### High precision mapping of replication origins

**Figure 3.**
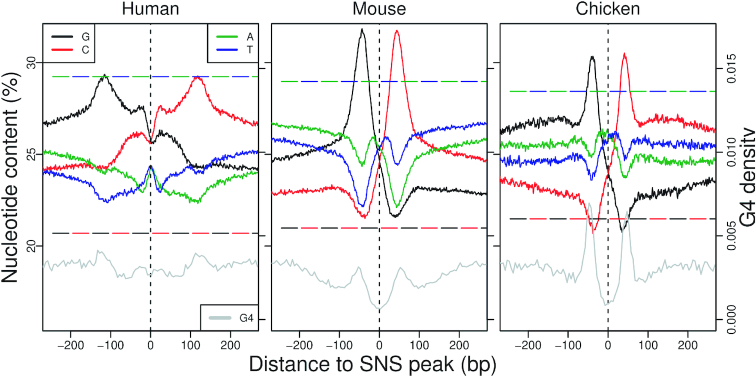
Replication origin sequence signature: Average nucleotide content in the 500 bp region centred on the SNS peak (plain line). Genome-wide average nucleotide contents are indicated by broken lines. Note that the 95% confidence interval were so small that they were not distinguishable when plotted on the same figure. Gray lines present the total number of G4 motifs found in all origins for 5 bp windows. To achieve a bp resolution for G4 motifs, we summarize each G4 motif by the position of its middle nucleotide.

To gain insights into the genetic elements controlling replication initiation, we analysed the average patterns of sequence composition and selective constraints along origins. One key point in these analyses is to define a relevant reference point to align origins. Here, we chose the SNS peak, i.e. the point of maximal SNS enrichment along the sequence (see Materials and Methods section). It has been previously shown that replication origins are associated with specific base composition skew profiles (17,32), likely resulting from the inversion of mutation patterns at the transition between leading and lagging strands (33,34). Interestingly, we observe that in the three species, both GC and AT-skew profiles ($S_{{\texttt {G}}{\texttt {C}}}=({\texttt {G}}-{\texttt {C}})/({\texttt {G}}+{\texttt {C}})$, $S_{{\texttt {A}}{\texttt {T}}}=({\texttt {A}}-{\texttt {T}})/({\texttt {A}}+{\texttt {T}})$) invert exactly at the SNS peak position (Figure 3) and extend over more than 2 kb on each side of the origin (Supplementary Figure S4). Our fine-scale analysis at kb resolution allowed us to map the position of maximal skew ∼ 50 bp to the SNS peak (compared with 280 bp identified in mouse (17)), suggesting that our method identifies the position of the replication start site very precisely. It should be noticed that the observed skew profiles result from mutations occurring in the germline. The fact that replication origins detected in somatic cells correspond precisely to skew inversions therefore indicates that many of them are also active in the germline. This is consistent with previous results indicating that a substantial fraction of replication origins (35–57%) are active in all examined cell types ((7) and Figure 6).

### Polymorphism data show evidence of selective pressure on replication origins

To investigate selective constraints impacting replication origins, we first analysed intraspecific sequence polymorphism. In humans, we used data from the 1000 genomes project (based on 2500 individuals), focusing on single nucleotide polymorphisms (SNPs) for which the nature of the ancestral and derived alleles could be inferred by comparison with great apes genomes (28).

To detect signatures of selection, we analysed SNPs with different derived allele frequencies (DAFs). Common variants (DAF > 10%) generally correspond to relatively old mutations that have spread in the population as a result of genetic drift, selection or GC-biased gene conversion (gBGC) (35). Rare variants (DAF < 1%) predominantly correspond to recent mutations, which are less affected by selection or gBGC and hence are expected to more accurately reflect the prevalence of mutational events (36). Rare variants and common variants constitute respectively 76.2% and 9.5% of the 84 million SNPs reported by the 1000 Genomes Project (28).

**Figure 4.**
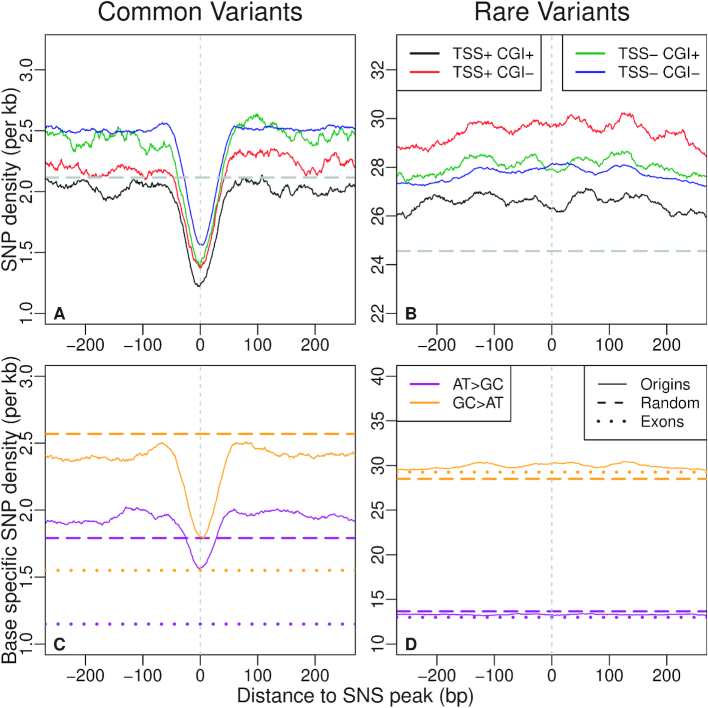
Density of genetic polymorphism around origins: average SNP density in the vicinity of all human SNS peaks (plain lines) for different classes of origins, depending on their association with CGI and/or TSSs. Genome-wide averages are indicated by broken lines. (**A**) Common variants (DAF > 10%). (**B**) Rare variants (DAF < 1%). (C, D) Base-specific SNP density (see Materials and Methods section) in the vicinity of all human SNS peaks (plain line). Broken lines: genome-wide average. Dotted line: average base-specific SNP density in all human coding exons. (**C**) Common A or T to C or G (${\texttt {A}}{\texttt {T}}>{\texttt {C}}{\texttt {G}}$) and C or G to A or T (${\texttt {C}}{\texttt {G}}>{\texttt {A}}{\texttt {T}}$) variants (DAF > 10%). (**D**) Rare variants (DAF < 1%).

To distinguish the effect of selection from that of gBGC, we separately analysed SNPs resulting from ${\texttt {G}}{\texttt {C}}\rightarrow {\texttt {A}}{\texttt {T}}\,$ mutations, ${\texttt {A}}{\texttt {T}}\rightarrow {\texttt {G}}{\texttt {C}}\,$ mutations or GC-conservative mutations. For rare variants, we observed that the SNP density is constant around SNS peaks and corresponds to the genome-wide average (Figure 4). This finding suggests that the mutation rate within origins does not differ from the rest of the genome. In contrast, we observed a strong deficit in common variants, specifically in the ∼40 bp region surrounding the SNS peak. This deficit is observed for all categories of SNPs, both GC→AT and AT→GC (Figure 4-(C-D)), or GC-conservative (Supplementary Figure S5), which implies that it is not caused by gBGC. Similarly, we observed a deficit of common indels (Supplementary Figure S5C and D) in the same 40 bp region. Hence, these observations demonstrate that the immediate vicinity of the SNS peak is subject to purifying selection. Many replication origins are associated with CGIs or TSSs, i.e. functional regulatory elements that are under selective pressure to control gene expression. Interestingly, the signature of selection around the SNS peak is observed for all categories of origins, irrespective of the presence of CGIs or TSSs (Figure 4A and B). Together with the observation that the SNP depletion occurs specifically in the immediate vicinity of the SNS peak, this result indicates that the signature of selection is directly linked to the function of replication initiation and not to the fact that origins are often associated with other functional elements.

Overall, the density in common SNPs within SNS peaks is 25% lower than in flanking regions (Figure 4-A). Although this deficit is weaker than the 40% depletion observed in coding exons (Figure 4C, dotted line), it clearly indicates that the sequence of human replication origins is subject to quite strong purifying selection. Hence, the ∼40-bp region centered on the SNS peak (hereafter referred to as the c"ore region of origins), includes genetic elements that are important for their function.

In chicken we observed a clear depletion of SNPs specifically in the core region as compared to the surrounding region (Supplementary Figure S6), which suggests that this region might be subject to purifying selection, as in humans. Unfortunately, polymorphism datasets currently available in mouse and chicken are based on relatively small population samples, which does not allow the comparison of rare and common variants. It is therefore not possible to determine to which extent the variation in SNP density along mouse and chicken origins are driven by mutational or selective effects.

### Selective pressure on replication origins is driven by sequence features that differ from G4 motifs

Previous analyses of human and mouse replication origins have revealed an enrichment in G4 motifs (6,17), and there is experimental evidence that these elements can contribute to their function (19). The highest density of G4 motifs is observed on each side of the replication origin (at about 150 bp of the SNS peak in human and 50 bp in mouse and chicken, Figure 3). However, the signal of selective pressure detected in humans is concentrated within a narrow 40 bp region centered on the SNS peak (Figure 4), which does not coincide with this maximal G4 motif enrichment. This result suggests that the selective pressure is not targeted towards G4 motifs and that the core region of origins probably contains another class of functional elements.

**Figure 5.**
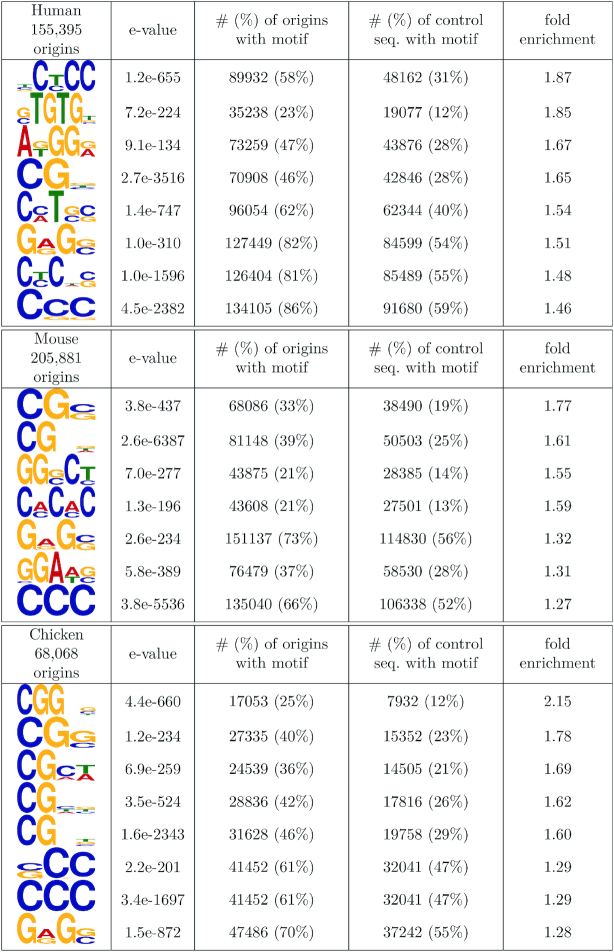
Motifs enriched in the 40 bp core region of origins for each species. Motifs were detected by adjusting the background model to compositional heterogeneities (see Materials and Methods). Control sequences were obtained by randomly sampling the same number of 40 bp regions in each genome.

To search for new sequence signatures in the core region of origins, we investigated motif enrichment using randomly selected segments as a control. Since the compositional heterogeneities of genomes strongly affects motif detection, the random segments were first clustered based on their kmer composition (5-mers) using *k*-means, hence providing clusters of homogeneous composition (see Materials and Methods and Supplementary Table S3), in which the motif search could be performed. We next annotated each core region of origins to a background cluster depending on their 5-mer contents and then searched for motifs cluster-wise to adjust the procedure to a heterogeneous background. Using the DREME software in the MEME suite (37), we selected motifs with strong e-values (≤1e^−200^) and further inspected motifs that were most enriched in the core region of origins compared with random segments. The detected motifs are all very short (≤6 bp), and strong enrichments are detected cluster-wise in motifs that are rich in CG dinucleotides for all species (Figure 5). The AC (GT) repeats previously identified in mouse (8) are confirmed and detected in human origins with 1.85-fold enrichment. Then, CCC(GGG) trinucleotides emerge as a shared sequence signature among species, with a two-fold cluster-wise enrichment in origins compared with random sequences. Since we show a relative depletion of G-quadruplex motifs within the core regions (Figure 3), these trinucleotides are likely to be distinct from flanking G-quadruplex motifs. Finally, the GAG (CTC) motif is another common feature between the three species. Consequently, our motif analysis highlights common short sequence features within the evolutionarily constrained core region of origins, with functional roles that remain to be experimentally investigated.

### Replication origins have experienced a rapid turnover in vertebrates

Given the selective constraints that we identified in humans, we further analyzed the conservation of replication origins on a larger evolutionary scale. We assessed the level of conservation at two levels. First, we investigated whether origins detected in chicken, human or mouse overlapped with conserved genomic segments (CGSs), identified in pairwise whole-genome alignments between these species (Table 1A). To estimate the number of overlaps expected by chance, we randomly sampled a set of segments in the mappable regions of the human genome with the same size and chromosomal distribution as origins (see Materials and Method).

**Table 1.** Analysis of functional conservation of origins across species. Schematic representation of **(A)** origins within conserved genomic segments (CGS) and **(B)** functionally conserved origins. **(C)** Origin conservation in vertebrates (among the top 25% most active origins). Human: ES-H9 cells, mouse: mESC, chicken: DT40 cell line. Top: Percentage of origins that overlap conserved genomic segments (CGS). Bottom: Percentage of functionally conserved origins. In parentheses: Conservation enrichment compared to the control experiment (see Randomization procedures in the Materials and Methods section for more details)

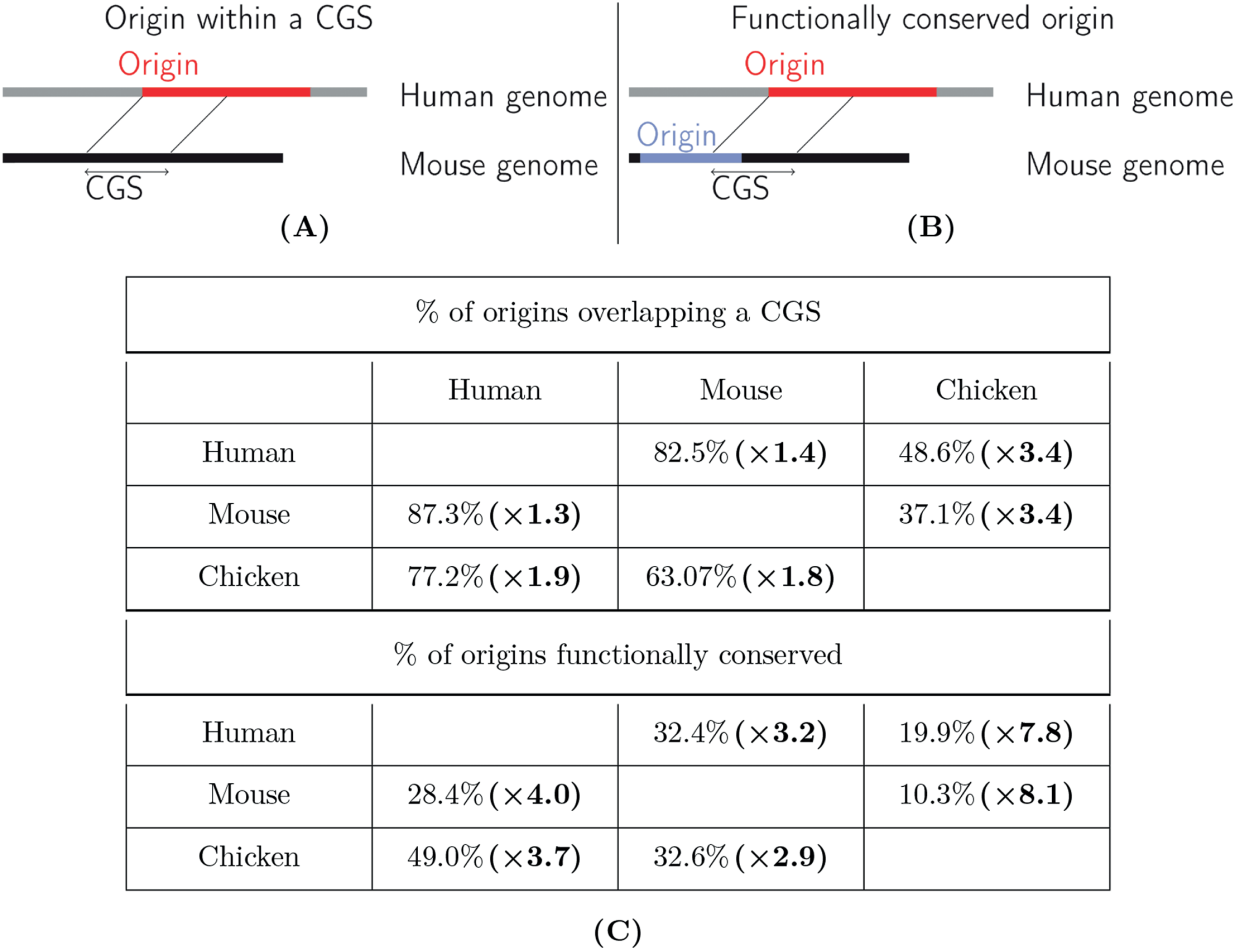

**Figure 6.**
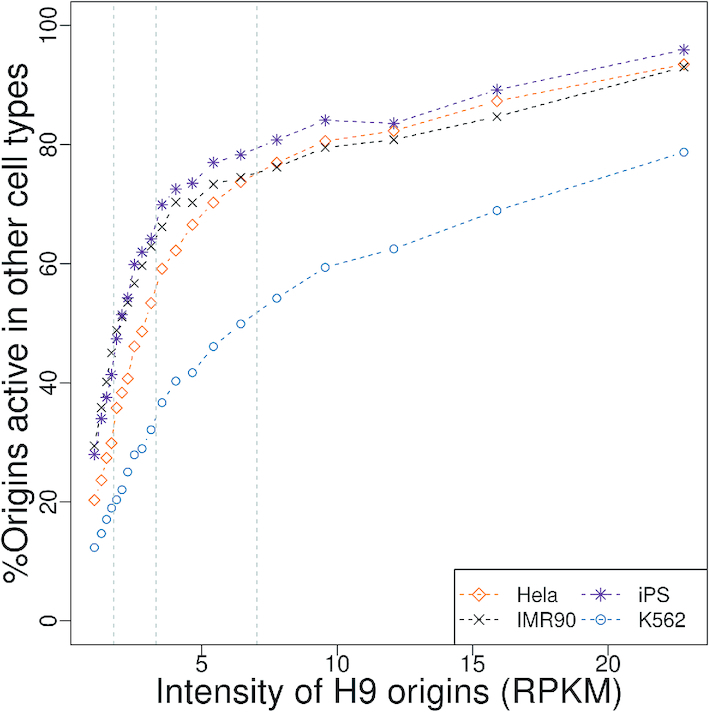
Fraction of human H9 origins that are active in four other human cell lines. Human H9 origins were categorized by increasing intensity (RPKM: number of SNS reads per kb, per million mapped reads). We grouped origins into 20 classes. For origins of a given class of intensity, we computed the percentage of origins of this category, that were also active in the other four cell lines. Grey dashed lines represent the 25, 50 and 75% origin activity quantiles.

Second, among origins detected in a given species that overlap a CGS, we determined whether they also correspond to active origins in the other species (Table 1-B). The evaluation of functional conservation across species raises two difficulties. First, the cell type studied in chicken differs from those analyzed in mammals and hence the functional conservation cannot be assessed for origins that are cell-type specific. Second, limitations in the sensitivity of origin detection, in particular for the less active origins, may lead to underestimate the level of functional conservation. To avoid these two biases, we focused our analysis on the top 25% most active origins. Indeed, we observed that among the most active human origins, >90% are detected in all cell-types (Figure 6), which implies (i) that the sensitivity of detection is very high for this subset of origins and (ii) most of them are constitutively active in all cell lines.

We found that 48.6% of human origins and 37.1% of mouse origins overlapped with chicken-mammal CGSs (Table 1). In both cases, these features correspond to a 3.5-fold enrichment compared to random expectation. In terms of functional conservation, 20% of human origins and 10.3% of mouse origins are functionally conserved in chicken (respectively 2.5% and 1.3% expected). Hence, only a small fraction of origins appears to be functionally conserved across vertebrates. Overall, human origins are 8-times more conserved in birds than expected by chance. For a comparison we analysed the conservation of TSSs of protein-coding genes: 50% of human TSSs overlap with chicken–human CGSs, among which 43% correspond to TSSs annotated in the chicken genome (Supplementary Table S5), which constitutes a 15-fold enrichment compared to random expectation. Thus, the conservation of human genetic elements in chicken is 2-times lower for replication origins than for TSSs. Even among mammals, the conservation of origins is quite limited: 83% of human origins overlap with human-mouse CGSs (60% expected by chance), but only 32.4% are active in mouse (Table 1). This constitutes a 3-fold enrichment, way below the 33-fold enrichment observed for TSSs (Supplementary Table S5).

**Figure 7.**
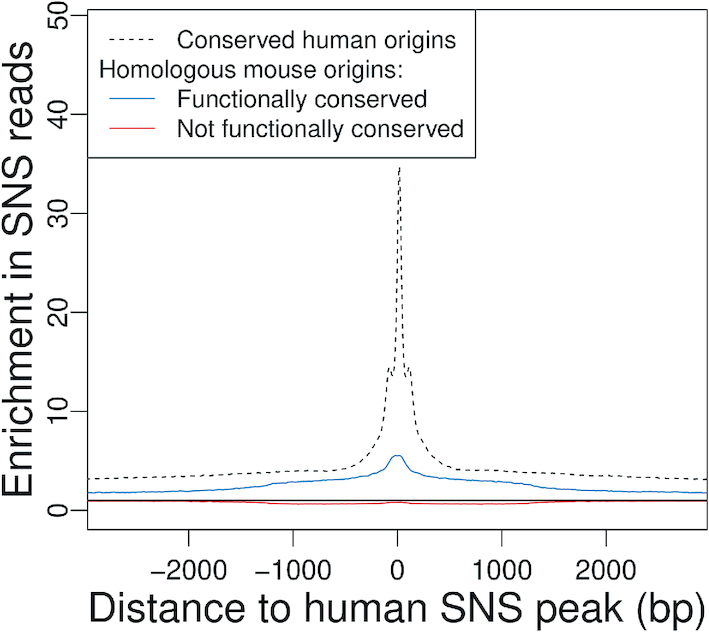
Conservation of replication origin activity at homologous loci. Average SNS read enrichment profile regions centered on the homolog of human SNS peaks in the mouse genome (plain line) compared to the genome-wide average, for conserved origins (*N* = 32 042) and non-conserved origins (*N* = 56 078). Dotted line: Average enrichment profile for all human origins overlapping a CGS, centered on the SNS peaks. Only origins with conserved SNS peaks are presented. Black plain line: Expected value without enrichment.

Finally, for the subset of human origins that were functionally conserved, we examined whether the SNS peak (i.e. the core region of origins, that is under selective pressure in human) corresponds to a zone of high SNS sequence read accumulation in mouse or chicken. As expected, we observe a read enrichment at the homologous position of the SNS peak (Figure 7). However, the strength of this peak is moderate, which indicates that even for origins with conserved activity, the precise position of the initiation starting point is not conserved. These observations are reproducible over all pairwise comparisons (Supplementary Figure S7). Overall, these observations indicate that despite evidence of selective pressure, the location of replication origins is poorly conserved, even on a small evolutionary scale (primate/rodent divergence).

### Long-term conservation of replication origins is driven by associations with functional elements

**Figure 8.**
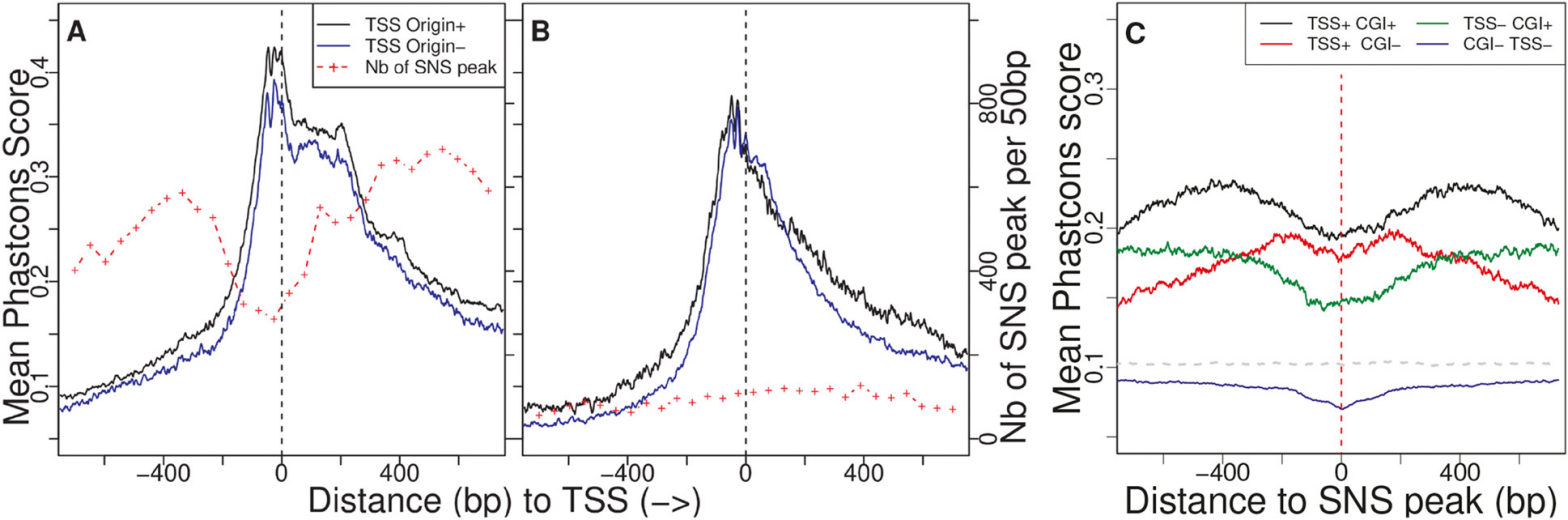
Profile of sequence conservation around TSSs and replication origins. (A, B) Human TSSs. Black: TSSs <750 bp away from an origin. Blue: TSSs that are not associated with an origin. The red dotted line presents the number of SNS peaks found around all TSSs (smoothed over 50 bp windows). All TSSs are oriented 5′ → 3′ relative to the transcription unit. (**A**) TSS associated with a CGI (**B**) TSS not associated to a CGI. (**C**) Average PhastCons scores calculated in a 1400 bp region centred on human SNS Peaks, depending on the origin association with TSSs and CGIs. The dashed line presents the genome-wide phastCons score average.

Mammalian replication origins are known to be frequently associated with TSSs and CGIs (5–8,15), and we found the same pattern in chicken (Supplementary Table S4). Interestingly, the fraction of mammalian origins that are functionally conserved in chicken is 2–3 times higher among origins associated with CGI and/or TSS compared to the other origins (Table 2 and Supplementary Table S6). However, the high conservation level of this subset of origins might simply reflect the selective pressure for promoter function. Alternatively, it is possible that there are specific functional constraints on the subset of promoters that possess replication origin activity. To test this latter hypothesis, we compared profiles of sequence conservation around human TSSs associated or not with an origin. We built these conservation profiles using PhastCons scores based on the multiple alignment of 46 vertebrate species (38). As expected, we found a strong and narrow peak of conservation immediately upstream of the TSS, both for promoters that overlapped a CGI and those that do not (Figure 8A and B). The average PhastCons scores tend to be slightly higher for promoters with origins than those without but the difference between the two scores is very weak compared to the 4-fold increase in PhastCons scores caused by the presence of TSSs. Moreover, the PhastCons score increase in TSSs associated with origins is independent of the presence of SNS peaks. Indeed, the density of SNS peaks varies substantially along CGI-containing promoters whereas the difference in PhastCons scores between promoters with or without origins remains constant (Figure 8A). Thus, the presence of an origin does not impact the level of conservation of promoters.

**Table 2.** Conservation of the 25% strongest human origins according to their association with TSSs and CGIs: percentage of origins overlapping conserved genomic segments, and percentage of functionally conserved origins

	Human–chicken		Human–mouse	
Origin category	% Overlapping a CGS	% Functionally conserved	Overlapping a CGS	% Functionally conserved
All	48.6% $\boldsymbol{(\times 3.4)}$	19.8% $\boldsymbol{(\times 7.8)}$	82.5% $\boldsymbol{(\times 1.4)}$	32.5% $\boldsymbol{(\times 3.2)}$
CGI[−] TSS[−]	33.8%	11.6%	75.2%	22.5%
CGI[+] TSS[−]	63.1%	29.9%	82.7%	36.5%
CGI[−] TSS[+]	58.4%	21.0%	92.2%	37.0%
CGI[+] TSS[+]	70.7%	33.4%	95.1%	52.4%
Dormant origins	11.4% $\boldsymbol{(\times 1.1)}$	-	63.1% $\boldsymbol{(\times 1.1)}$	-

Dormant origins are defined as HeLa MCM7 Chip-Seq peaks (23) that do not overlap HeLa origins detected by SNS-seq. Functional conservation could not be assessed for dormant origins since we only have data on the human HeLa cell line (and not for chicken nor mouse).

We also examined sequence conservation profiles, centred on SNS peaks of human origins (Figure 8C). As expected, replication origins that collocate with TSSs or with CGIs have higher PhastCons scores than those that do not. However, in all cases, the SNS peak position is, on average, less conserved than its immediate surroundings. The same patterns are observed in mouse (Supplementary Figure S8). Thus, all these observations indicate that, on the evolutionary scale considered herein, the conservation of replication origins solely results from their association with functional elements (CGIs, TSSs) that are under strong selective pressure to be conserved.

## DISCUSSION

Despite the central role of DNA replication in the life cycle of living organisms, the molecular mechanisms inducing the firing of replication origins are still poorly understood. To track evolutionary signatures that could unravel sequence features controlling replication initiation, we conducted the first comparative analysis of high-resolution maps of replication origins in chicken, human and mouse. Our analyses rely on published and original data from short nascent strand sequencing, a strategy for the fine mapping of replication origins that has been validated by other independent high-throughput protocols (7,23,24). Our analyses show the reliability of such detections, since more than 90% of the most active origins (intensity >7.5 RPKM, Figure 6) in the H9 human, cell lines are also detected in other datasets from different cells and different laboratories. Based on these detections, our meta-analysis first showed that large-scale (100 kb) replication landscapes have been largely remodeled during the evolution of vertebrates. As expected, given the differences in genome sizes, the number of origins is much higher in mammals than in chicken. However, replication landscapes are not simply driven by genome size. In particular, the mouse genome is characterized by a high density of origins of relatively low intensity compared to the two other species (Figure 2). Thus, whereas the replication initiation activity in human and chicken genomes is concentrated in clusters of very active loci, the mouse genome presents a more uniform distribution. Interestingly, this shift in replication landscapes coincides with differences in large-scale (100 kb) GC-content landscapes (the so-called isochores), which are much more uniform in mouse than in human or chicken (see (39,40) and Supplementary Figure S3).

The analysis of genetic polymorphism within human origins revealed a clear signature of purifying selection, specifically in the core region of origins, a narrow region (∼40 bp) surrounding the SNS peak, which probably corresponds to the replication initiation site. In the three species, there is a strong excess of origins associated with TSSs or CGIs, which suggests that these genetic elements constitute a favorable context for initiating replication. However, the selection signal on SNS peaks in human is not driven by functional constraints on TSSs or CGIs since it is observed irrespective of the presence of such elements (Figure 4). This selective pressure therefore probably results from the presence of specific signals involved in the initiation of replication. Our motif detection procedure revealed an enrichment of CG dinucleotides, CCC  triplets and GAG in the core region of origins. Interestingly, this enrichment is observed in all three species. However, these motifs are very short, and hence are also abundant outside of replication origins. This core region must therefore contain another type of signal that does not correspond to simple sequence motifs – for instance, a structural signal. The nature of this signal and its functional role in replication initiation remain to be described. Our analysis also highlighted the heterogeneities of sequence features characterizing this very specific region, as previously shown in mouse origins (8), hence indicating the potential existence of different classes of origins driven by distinct combinations of DNA binding factors (8,41). However, the causes of the observed selective pressure remain enigmatic and might reflect constraints on the local structure of the DNA, rather than on sequence motifs (10). Intriguingly, we showed that this selective pressure is *not* due to the presence of G4 motifs, which tend to be located further upstream of the SNS peak (Figure 3). There is evidence that G4 motifs are functionally important, but their presence is not sufficient to initiate replication (19).

One striking result of the present analysis is that despite this evidence of purifying selection, which is clearly visible in human polymorphism datasets, inter-species comparisons revealed very limited conservation of replication origins on larger evolutionary scales. Only 10–20 % of mammalian origins are functionally conserved in chicken, and 30% of human origins are functionally conserved in mouse. The number of functionally conserved origins ranged from three to eight times higher than expected by chance, but this excess can be explained, in large part, by the strong association of origins with other functional elements (TSSs or CGIs) that are themselves conserved in vertebrates (Table 2 and Supplementary Table S6). Furthermore, among the small subset of origins that are functionally conserved, the precise location of SNS peaks is not conserved (Figure 7 and Supplementary Figure S7). All these observations support a rapid turnover of replication start sites during evolution.

A recent study of mini chromosome maintenance (MCM) complexes in HeLa cells showed that, in addition to active replication origins, the human genome contains a large number of dormant origins, which have been proposed to serve as a backup system to maintain genome integrity during replication stress (23). We analyzed this dataset of dormant origins, and found that their level of sequence conservation is much lower than that of active origins (Table 2), which suggests that functional constraints are even weaker for dormant origins.

One possible hypothesis to reconcile the contrasting patterns of conservation observed on different time-scales is a selective pressure to maintain a sufficient density of replication origins along chromosomes, but no constraint on the precise location of these origins. According to this model, if the number and intensity of origins in a given chromosome are just sufficient to ensure its proper replication, any mutation that would disrupt an origin would be counter-selected. However, a mutation that creates a new origin might occasionally occur (e.g. by converting a dormant origin into a firing origin). Such a mutation would be neutral and hence could be fixed by random drift. Then, once one additional origin is fixed, mutations that would disrupt a previously existing origin would be neutral. Thus, this model would explain why replication origins appear to be under purifying selection on a short time-scale but not conserved over the long term. A similar model has recently been proposed to explain the evolution of yeast replication origins (42). This peculiar temporal conservation pattern is not specific to replication origins: for many functional elements located in non-coding regions, signatures of selection are better revealed by analysis of polymorphism than by inter-species comparisons (43).

The high plasticity of the spatial program of replication is also surprising in light of the evidence showing that the temporal replication program is organized into large 1 Mb-long domains, which are well conserved in mammals (44,45). This discrepancy could imply that the temporal and spatial program of replication evolve independently. Alternatively, the observation that replication origin density is high in early replication domains and low in late replicating ones could lead to another explanation. Indeed, if origin losses are replaced by origin gains in close proximity, as suggested in a recent study in yeast (42), the origin density of replication domains—and hence, their replication timing—would be maintained despite the rapid turnover of origins.

In conclusion, our study highlights the flexibility of the spatial program of replication in vertebrate genomes, unraveling the existence of a novel genetic determinant of replication origins in vertebrates, the precise nature of which remains to be determined.

## DATA AVAILABILITY

The high-throughput sequencing data from this study have been submitted to the NCBI Gene Expression Omnibus (GEO; http://www.ncbi.nlm.nih.gov/geo/) under accession number GSE119488. Processed Data resulting from the meta-analysis of replication origins are available on GitHub (https://github.com/Flomass/EvolutionReplicationOrigins) together with dedicated scripts to perform the analysis described herein.

## Supplementary Material

gkz182_Supplemental_Files
