# Supplementary material for: Evolution of replication origins in vertebrate genomes: rapid turnover despite selective constraints
Source: Nucleic Acids Res. 2019 Mar 27;47(10):5114–25. doi: 10.1093/nar/gkz182 (PMC6547456; doi:10.1093/nar/gkz182)
Supplement: gkz182_Supplemental_Files [file gkz182_supplemental_files.pdf]

# SUPPLEMENTARY MATERIAL

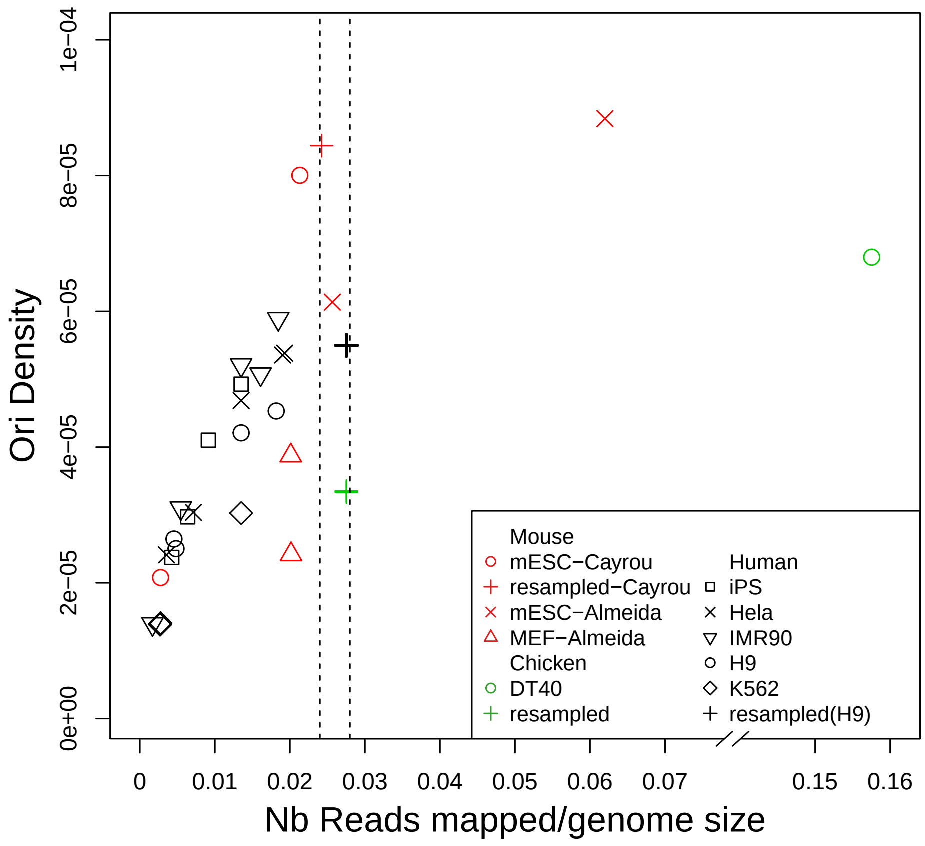


**Figure S1.** Origins density increases with sequencing depth. Origins density was calculated as the ratio of the total number of origins detected in a dataset divided by the size of the mappable part of the genome. Vertical dashed lines correspond to the coverage of the datasets used to conduct the evolutionary analysis.

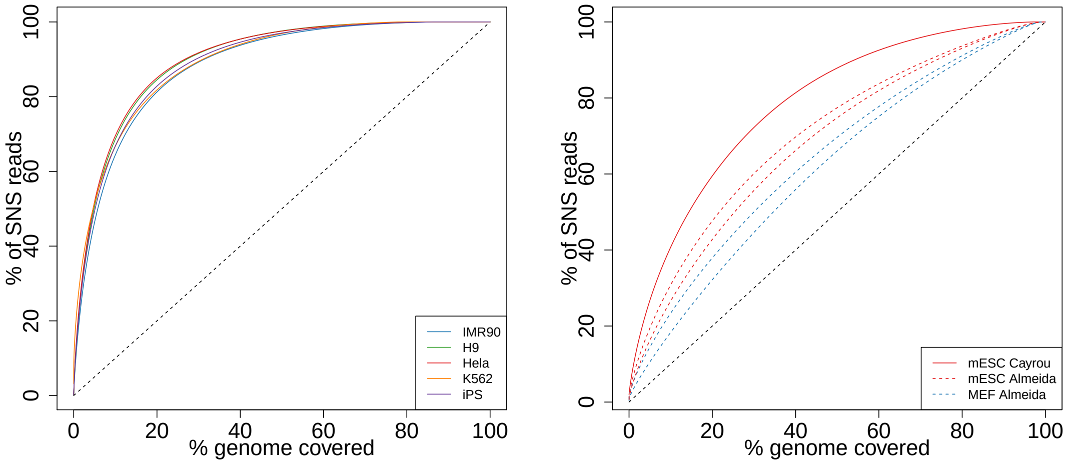


**(A)** **(B)**

**Figure S2.** Replication initiation landscapes in different cell types and for datasets generated in different laboratories: Cumulative proportion of SNS reads mapping into the $x$ richest 100 kb regions of the genomes for SNS experiment performed in different cell lines and/or different laboratories. The black dotted line corresponds to the expectation if reads were distributed randomly over the genome. **(A)** For various human cell types. **(B)** For various mouse cell types and various replicates of the same experiments realized in two different laboratories.

| Sample Id | Number of mapped reads | Number of Origins detected | % of Genome Covered | Mean origin length |
|---|---|---|---|---|
| *Chicken, DT40* | | | | |
| SRX4643655 | 173,295,362 | 68,068 | 6.0% | 884 |
| *Mouse, mESC* | | | | |
| *(Cayrou et al.)* | | | | |
| SRR2000505 | 43,870,450 | 203,809 | 5.4% | 677 |
| SRR2000507 | 5,676,763 | 52,913 | 1.4% | 662 |
| Overlap | | 35,545 (67%) | | |
| *(Almeida et al.)* | | | | |
| SRR5650899 | 52,770,039 | 156,242 | 3.9% | 660 |
| SRR5650900 | 127,560,711 | 225,251 | 5.9% | 689 |
| Overlap | | 129,015 (82%) | | |
| *Mouse, MEF* | | | | |
| *(Almeida et al.)* | | | | |
| SRR5650895 | 41,459,153 | 61,591 | 1.4% | 612 |
| SRR5650896 | 41,383,367 | 98,656 | 2.5% | 656 |
| Overlap | | 46286 (75%) | | |
| *Human,H9* | | | | |
| SRR494093 | 10,965,801 | 74,783 | 2.1% | 787 |
| SRR494094 | 43,875,325 | 128,090 | 3.6% | 795 |
| SRR494095 | 11,636,781 | 70,766 | 1.9% | 742 |
| Overlap | | 48,348 (64%) | | |
| *Human, Hela* | | | | |
| SRR494099 | 8,571,107 | 68,204 | 1.8% | 735 |
| SRR494100 | 45,915,571 | 151,396 | 3.9% | 735 |
| SRR494101 | 46,607,229 | 152,159 | 4.0% | 734 |
| SRR494102 | 17,238,919 | 85,870 | 2.4% | 804 |
| Overlap | | 63,730 (93%) | | |
| *Human, IMR90* | | | | |
| SRR494089 | 4,067,936 | 39,427 | 1.0% | 716 |
| SRR494090 | 44,539,716 | 166,496 | 4.3% | 736 |
| SRR494091 | 13,173,025 | 87,690 | 2.3% | 742 |
| SRR494092 | 38,851,752 | 143,440 | 3.8% | 750 |
| Overlap | | 30,953 (78%) | | |
| *Human, iPS* | | | | |
| SRR494096 | 22,033,477 | 115,935 | 3.0% | 732 |
| SRR494097 | 10,250,855 | 67,103 | 1.8% | 751 |
| SRR494098 | 15,371,008 | 84,020 | 2.2% | 747 |
| Overlap | | 49,116 (73%) | | |
| *Human, K562* | | | | |
| SRR830646 | 6,442,105 | 39,300 | 1.1% | 819 |
| SRR830647 | 6,662,962 | 39,755 | 1.2% | 826 |
| SRR830648 | 6,512,929 | 39,096 | 1.1% | 825 |
| SRR830649 | 6,510,424 | 39,386 | 1.2% | 827 |
| SRR830650 | 6,455,368 | 39,248 | 1.1% | 825 |
| Overlap | | 20,861 (53%) | | |

**Table S1.** Caption on the next page

**Table S1.** Summary of the SNS data meta-analysis. The overlap is defined as the intersection of all detections for a given cell line. The percentage of overlap is the number of overlapping origins divided by the smallest number of detected origins among replicates.

| Sample Id | Number of mapped reads | Coverage (Reads per kb) | Number of Origins detected | % of Genome Covered by Origins | Mean origin length |
|---|---|---|---|---|---|
| *Chicken, DT40* sample | 30,276,092 | 28 | 33,489 | 3.0% | 897 |
| *Mouse, mESC (Cayrou et al.)* SRR2000505-7 | 49,859,260 | 24 | 205,881 | 5.5% | 690 |
| *Human,H9* SRR494093-4-5 | 66,477,907 | 28 | 155,395 | 4.3% | 791 |

**Table S2.** Summary of the SNS data used to conduct the evolutionary analysis. "Sample" for the DT40 corresponds to a subsampling of chicken SNS data to reach comparable sequencing depth as compared with other species. We performed 3 independent subsamplings, and checked that 80% of origins detected in a subsample were confirmed by detections in the original dataset of 68,068 origins. The SRR494093-4-5 sample is the combination of SRR49093, SRR49094 and SRR49095, and SRR2000505-7 the combination of samples SRR2000505 and SRR2000507.

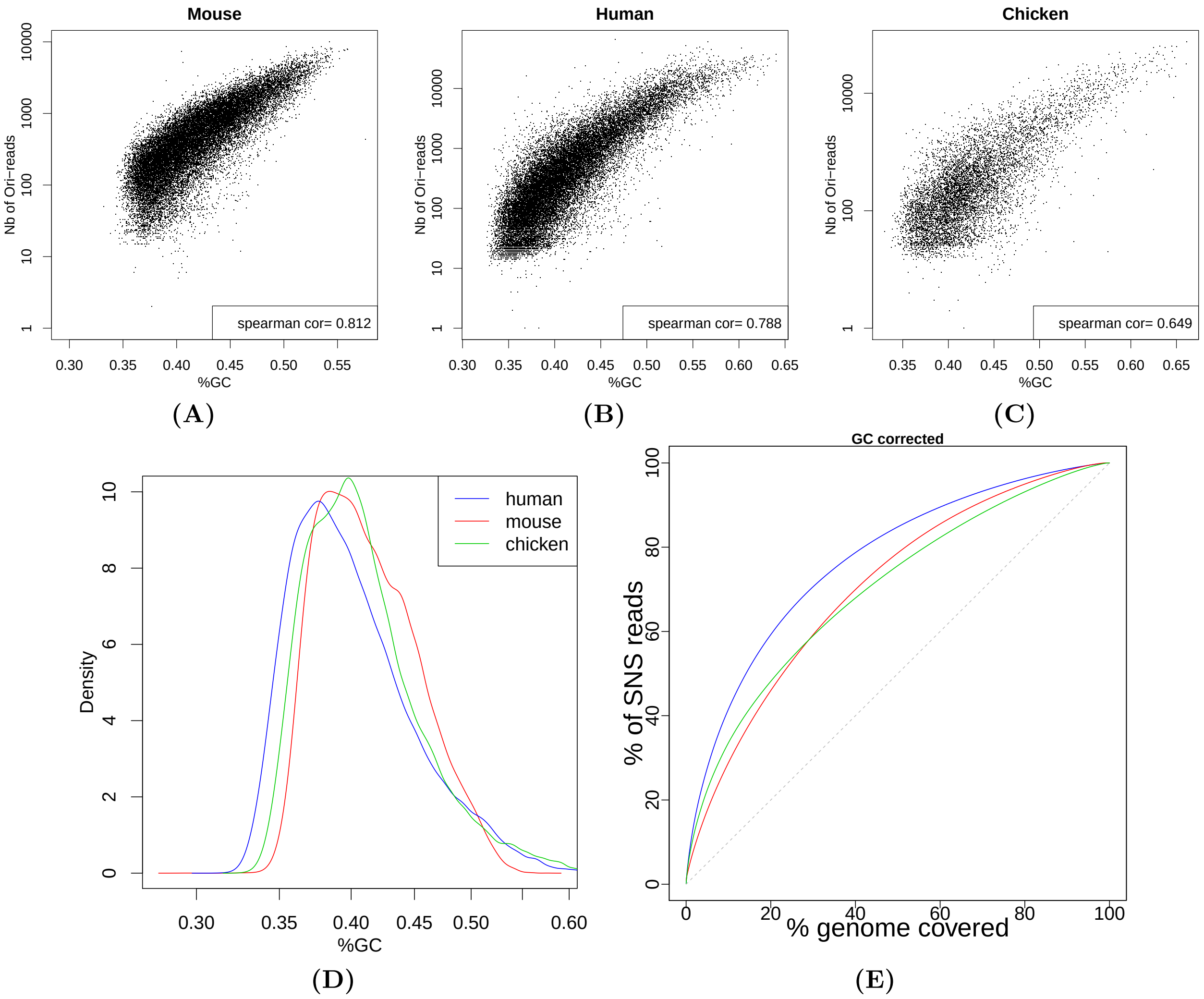


**Figure S3.** Impact of GC content variations on Replication intiation landscapes. **(A)-(C)**: Correlation between GC content and the number of SNS reads mapping into an origin in 100 kb windows in the mouse **(A)** human **(B)** and chicken **(C)** genomes. **(D)** Distribution of the regional GC content calculated on 100 kb windows. **(E)** Cumulative proportion of SNS reads mapping into the $x$ richest 100 kb regions of the genomes after correcting for GC bias. Windows of the human and chicken genomes were sampled to match their GC content with the GC content of the mouse genome.

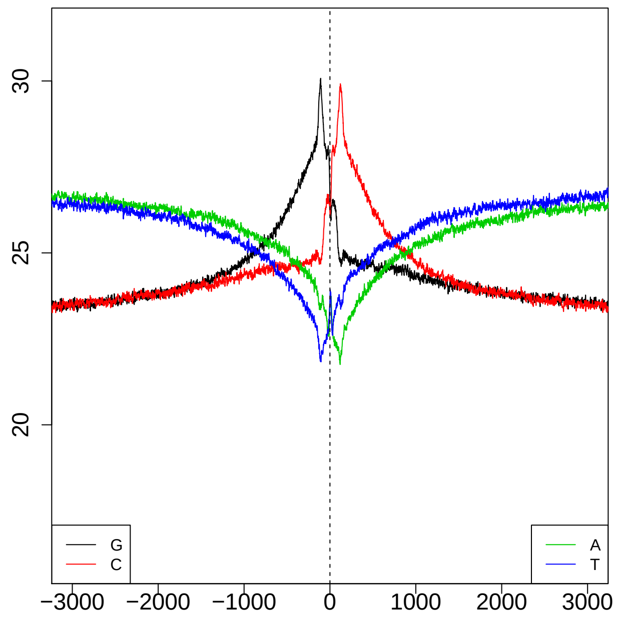


**Figure S4.** Extended replication origin sequence signature in human H9: Average nucleotide content in the 3000 bp region centred on the SNS peak (plain line). Genome-wide average nucleotide contents are indicated by broken lines. Note that the 95% confidence interval were so small that they were not distinguishable when plotted on the same figure. Grey lines present the total number of G4 motifs found in all origins for 5 bp windows. To achieve a bp resolution for G4 motifs , we summarize each G4 motif by the position of its middle nucleotide.

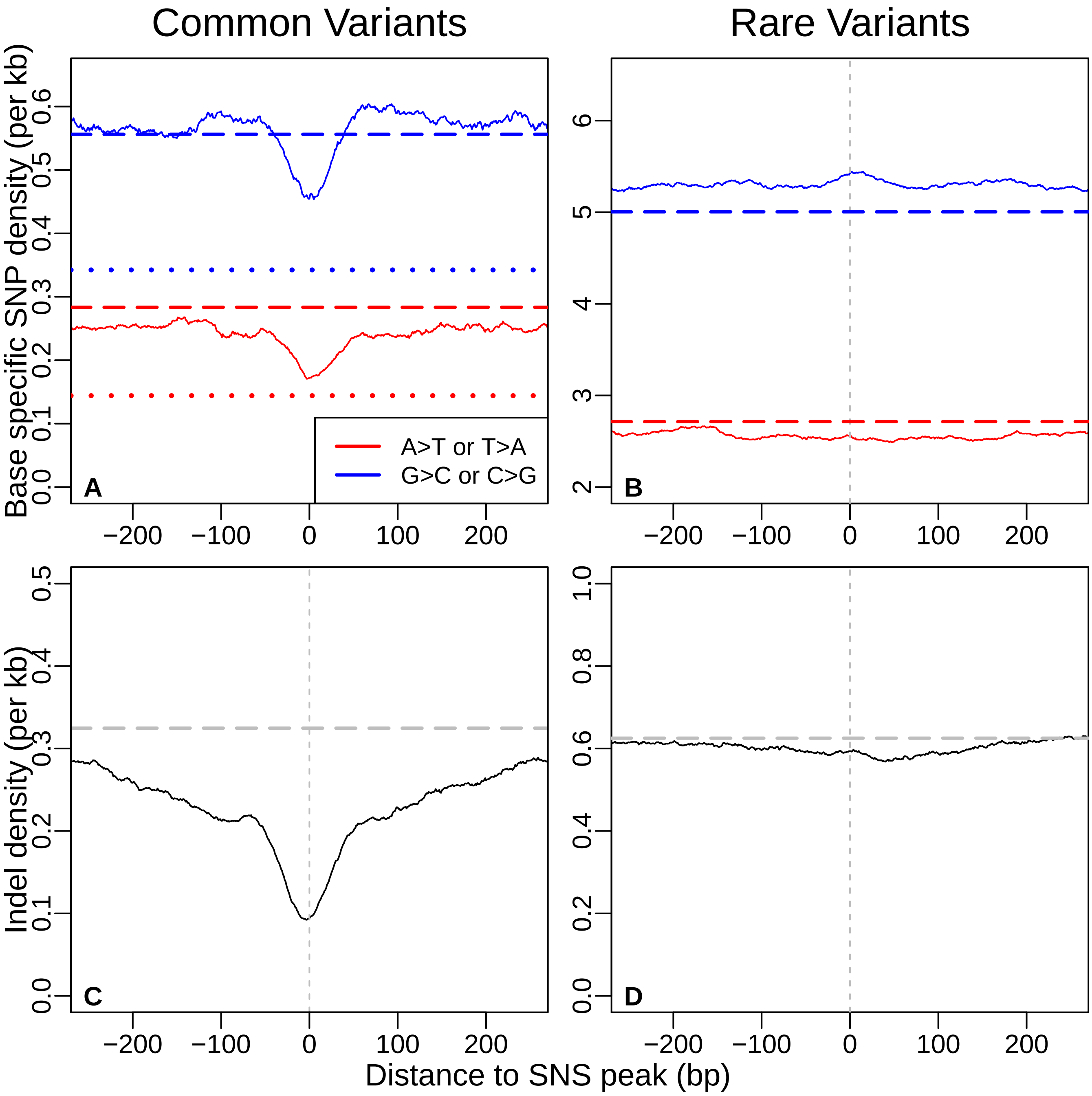


**Figure S5.** Density of genetic polymorphsism around origins. Base-specific SNP density (see Methods section) in the vicinity of all human SNS-peaks (plain line), in random genomic segments (broken lines) or in coding exons (dotted line). **(A)** Common AT→AT or GC→GC variants ($DAF < 1\%$) **(B)** Rare AT→AT or GC→GC variants ($DAF > 10\%$). **(C-D)** Indel density in the vicinity of human SNS-peaks (plain line) or random genomic segments (broken line). **(C)** Common Indels ($DAF < 1\%$). **(D)** Rare Indels ($DAF > 10\%$).

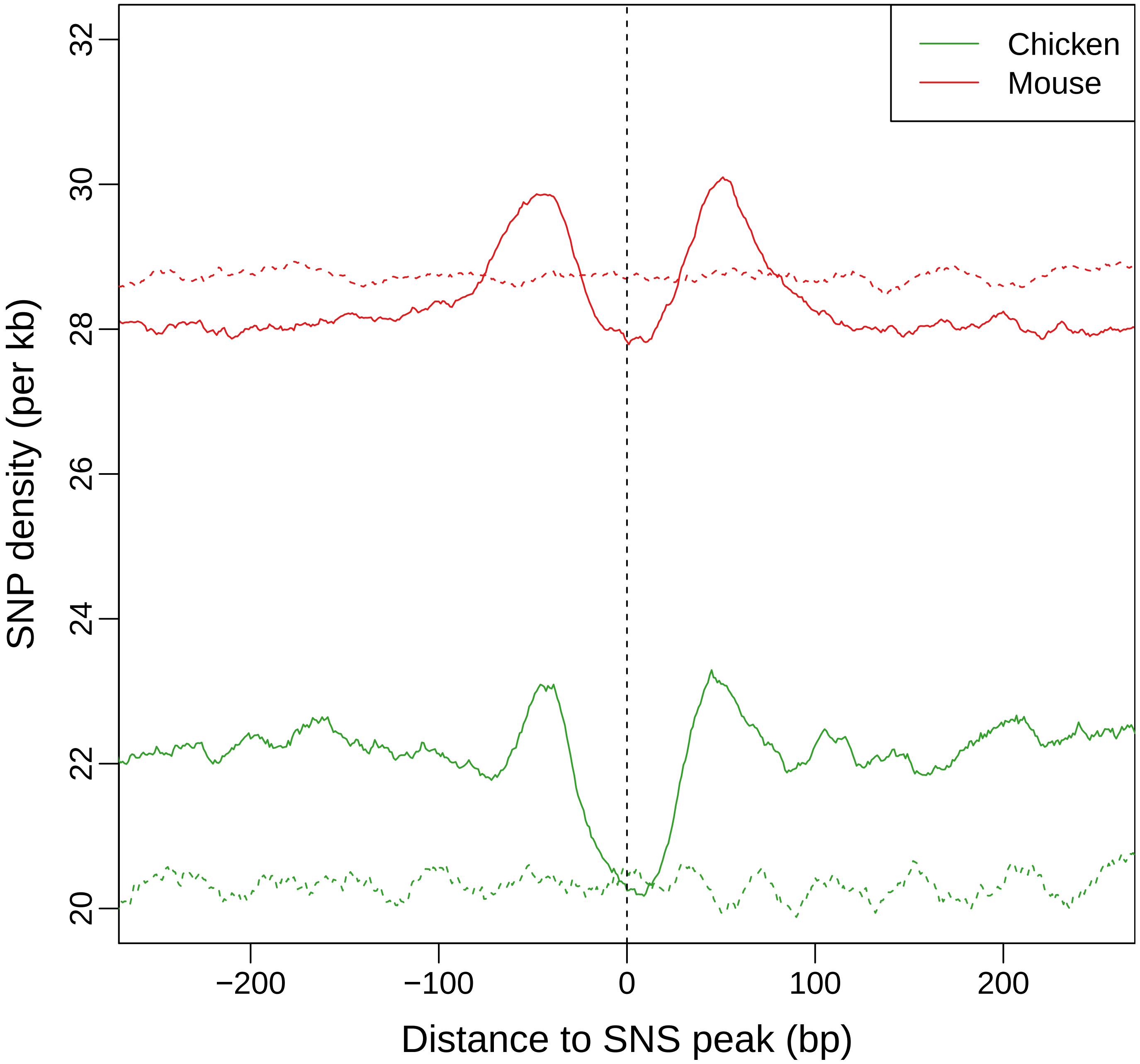


**Figure S6.** Density of genetic polymorphsism around mouse and chicken origins. Average SNP density in the vicinity of the SNS-peaks in mouse and chicken (plain lines). Broken lines indicate the genome-wide averages in each species.

| cluster | kmer frequency logo | # rand segments in cluster | # oris in cluster |
|---|---|---|---|
| Human | | | |
| 1 | 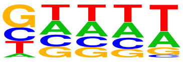 | 24853 | 4126 |
| 2 | 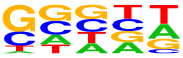 | 23825 | 12158 |
| 3 | 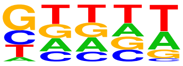 | 32643 | 88254 |
| 4 | 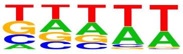 | 22443 | 7558 |
| 5 | 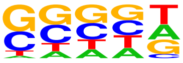 | 23709 | 4389 |
| 6 | 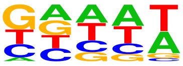 | 27733 | 38910 |
| Mouse | | | |
| 1 | 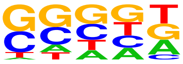 | 34014 | 25862 |
| 2 | 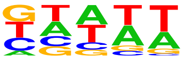 | 34386 | 34088 |
| 3 | 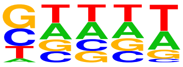 | 33445 | 20871 |
| 4 | 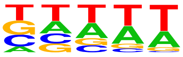 | 38084 | 92151 |
| 5 | 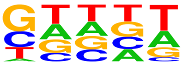 | 31841 | 15762 |
| 6 | 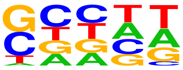 | 33360 | 17147 |
| Chicken | | | |
| 1 | 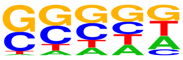 | 8028 | 0 |
| 2 | 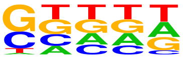 | 10141 | 2050 |
| 3 | 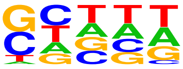 | 10515 | 3029 |
| 4 | 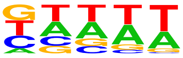 | 14704 | 29247 |
| 5 | 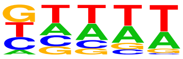 | 11690 | 10787 |
| 6 | 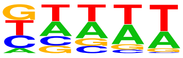 | 12868 | 22955 |

**Table S3.** Kmers (5-mers) composition of background clusters. Clusters are inferred on randomly selected segments and origins are assigned to the background clusters based on their composition (see Methods).

| Species | G-quadruplex | CGI | TSS |
|---|---|---|---|
| Human | 32% (9.2%) | 13% (2.8%) | 11.3% (3.6%) |
| Mouse | 24.8% (11.3%) | 8.9% (1.8%) | 9.2% (2.1%) |
| Chicken | 42.1% (10.1%) | 39.6% (6.7%) | 10.2% (2.5%) |

**Table S4.** Association of origins with genomic elements. Percentage of origins that overlap, a TSS, a CGI or a G-quadruplex motif in human, mouse or chicken genomes. Figures in parenthesis give the expected value for random segments. For details on the randomization procedure, see the Method section.

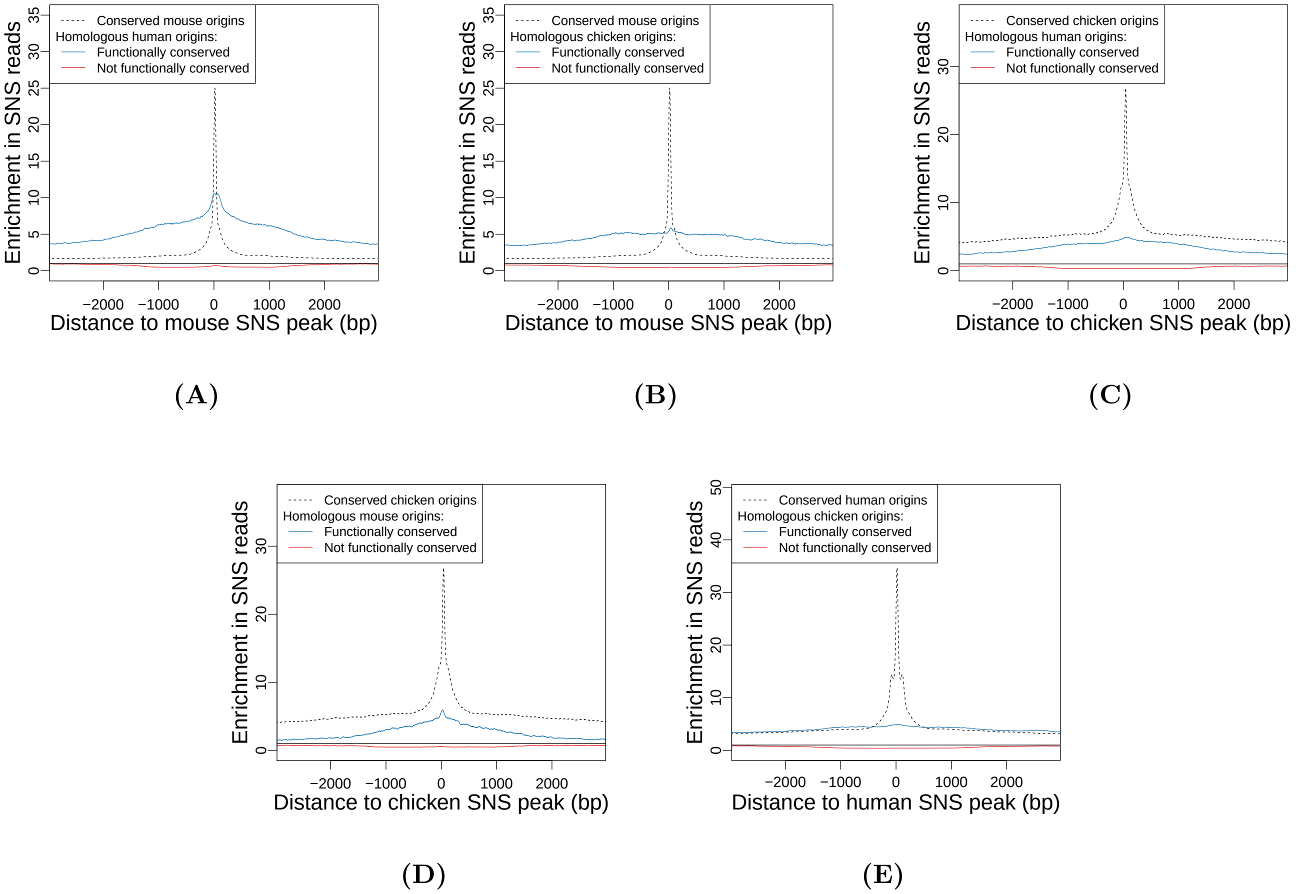


**Figure S7.** Conservation of replication origin activity at homologous loci for all pairwise comparisons. See Fig. 7 for details. **(A)** Mouse origins (broken line), and the homologs of mouse origins in the human genome; **(B)** Mouse origins (broken line), and the homologs of mouse origins in the chicken genome; **(C)** Chicken origins (broken line), and the homologs of chicken origins in the human genome; **(D)** Chicken origins (broken line), and the homologs of chicken origins in the mouse genome. **(E)** Human origins (broken line), and the homologs of human origins in the chicken genome.

| % of TSSs overlapping a CGS | | | |
|---|---|---|---|
| | Human | Mouse | Chicken |
| Human | | 93.4% (×**1.6**) | 50.4% (×**4.2**) |
| Mouse | 94.1% (×**1.5**) | | 39.8% (×**4**) |
| Chicken | 47.1% (×**1.6**) | 41.6% (×**1.6**) | |
| **% of TSS functionally conserved** | | | |
| Human | | 37.4% (×**33.6**) | 21.5% (×**15.4**) |
| Mouse | 42.2% (×**35.6**) | | 17.5% (×**20.7**) |
| Chicken | 17.5% (×**8.1**) | 12.8% (×**11.6**) | |

**Table S5.** Conservation of TSSs accross species. Top: Percentage of TSSs that overlap conserved genomic segments (CGS). Bottom: percentage of functionally conserved TSSs (see Method for details). In parenthesis: conservation enrichment compared to the control experiment (see Randomization procedures in the Method section for more details).

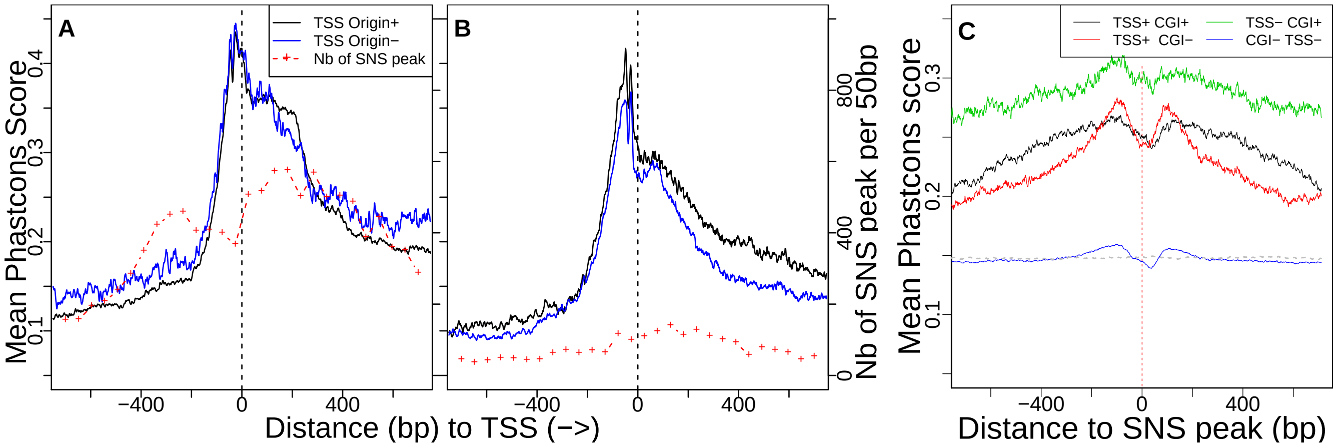


**Figure S8.** Profile of sequence conservation around TSSs and replication origins in mouse. **(A-B)** Mouse TSSs. Black: TSSs less than 750 bp away from an origin. Blue: TSS that are not associated with an origin. The red dotted line presents the number of SNS peaks found around all TSSs (smoothed over 50 bp windows). All TSSs are oriented $5' \rightarrow 3'$ relative to the transcription unit. **(A)** TSS associated with a CGI **(B)** TSS not associated with a CGI. **(C)** Average PhastCons scores calculated on a 1400 bp region centred on human SNS Peaks depending on the origin association with TSSs and CGIs. The dashed line presents the genome-wide phastCons score average.

| | Mouse - Chicken | | Mouse - Human | |
|---|---|---|---|---|
| All | 37.1% (×**3.4**) | 10.3% (×**8.1**) | 87.3% (×**1.3**) | 28.5% (×**4.0**) |
| CGI[-] TSS[-] | 25.9% | 4.9% | 82.2% | 20.4% |
| CGI[+] TSS[-] | 59.9% | 25.3% | 94.9% | 45.0% |
| CGI[-] TSS[+] | 52.7% | 12.0% | 95.6% | 35.8% |
| CGI[+] TSS[+] | 61.4% | 23.5% | 98.7% | 47.6% |
| | **Chicken - Mouse** | | **Chicken - Human** | |
| All | 63.1% (×**1.8**) | 32.6% (×**2.9**) | 77.3% (×**1.9**) | 49.0% (×**3.7**) |
| CGI[-] TSS[-] | 51.4% | 22.4% | 64.4% | 37.7% |
| CGI[+] TSS[-] | 62.4% | 31.6% | 78.8% | 49.6% |
| CGI[-] TSS[+] | 66.7% | 33.6% | 80.1% | 52.3% |
| CGI[+] TSS[+] | 74.7% | 44.1% | 86.2% | 58.0% |

**Table S6.** Conservation of the 25% strongest human origins according to their association with TSSs and CGIs: Percentage of origins overlapping conserved genomic segments, and percentage of functionally conserved origins.
